# Pharmacological induction of translational readthrough of nonsense mutations in the retinoblastoma (RB1) gene

**DOI:** 10.1371/journal.pone.0292468

**Published:** 2023-11-02

**Authors:** Mireia Palomar-Siles, Viktor Yurevych, Vladimir J. N. Bykov, Klas G. Wiman

**Affiliations:** Department of Oncology-Pathology, BioClinicum, Karolinska Institutet, Stockholm, Sweden; University of Ferrara, ITALY

## Abstract

The retinoblastoma protein (Rb) is encoded by the *RB1* tumor suppressor gene. Inactivation of *RB1* by inherited or somatic mutation occurs in retinoblastoma and various other types of tumors. A significant fraction (25.9%) of somatic *RB1* mutations are nonsense substitutions leading to a premature termination codon (PTC) in the *RB1* coding sequence and expression of truncated inactive Rb protein. Here we show that aminoglycoside G418, a known translational readthrough inducer, can induce full-length Rb protein in SW1783 astrocytoma cells with endogenous R579X nonsense mutant *RB1* as well as in MDA-MB-436 breast carcinoma cells transiently transfected with R251X, R320X, R579X or Q702X nonsense mutant *RB1* cDNA. Readthrough was associated with increased *RB1* mRNA levels in nonsense mutant *RB1* cells. Induction of full-length Rb protein was potentiated by the cereblon E3 ligase modulator CC-90009. These results suggest that pharmacological induction of translational readthrough could be a feasible strategy for therapeutic targeting of tumors with nonsense mutant *RB1*.

## Introduction

The retinoblastoma (*RB1*) tumor suppressor gene encodes the Rb protein that controls cell cycle progression in the G1 phase [1]. Specifically, hypophosphorylated Rb binds to the E2F family of proteins, thereby preventing the transcription of genes required for S phase progression. Rb hyperphosphorylation caused by cyclin D-CDK4/6 leads to disruption of Rb-E2F binding, allowing the cell to proceed through S phase [2]. The Rb protein is also involved in other cellular processes such as organization of chromosomal domains and protection of genomic stability [3–5].

According to the Catalogue Of Somatic Mutations In Cancer (COSMIC) database, 25.9% of all somatic *RB1* mutations correspond to nonsense substitutions [6]. Moreover, 34.1% of reported *RB1* mutations in the Leiden Open Variation Database (LOVD) (http://rb1-lsdb.d-lohmann.de/) are nonsense mutations; this database includes both somatic and germline mutations. The most common *RB1* nonsense somatic mutations are R251X, R579X and R320X [6]. Nonsense mutations introduce premature termination codons (PTCs) in the mRNA. Ribosomes stall at the PTC and subsequent binding of release factors eRF1 and eRF3 causes the release of a truncated and non-functional protein [7]. In addition, PTC-containing mRNAs are usually present at low levels in cells due to nonsense-mediated decay (NMD) [8].

Expression of full-length protein from a nonsense mutated gene can be achieved by induction of translational readthrough [7]. Aminoglycosides such as G418 and Gentamicin have been shown to induce readthrough of nonsense mutations in cancer-relevant genes, e.g. *TP53* [9, 10], *BRCA1* [11], and *PTEN* [12]. However, the clinical utility of aminoglycosides such as G418 is limited by their reported ototoxicity [13] and nephrotoxicity [14]. Recent studies have identified compounds that potentiate the readthrough effect of G418, such as the cereblon E3 ligase modulator CC-90009 that promotes proteasomal degradation of eukaryotic release factor eRF3 [15].

Our main aim in this study was to examine if the aminoglycoside G418 can induce translational readthrough of nonsense mutations in the *RB1* gene. In addition, taking into account the toxicity of G418 at the doses required to induce readthrough, we wished to test combination treatment with possible potentiators of readthrough to enhance levels of full-length Rb protein. Here we show that G418 induces translational readthrough of *RB1* nonsense mutants R251X, R320X, R579X and Q702X, and that combination of G418 with CC-90009 further increases levels of full-length Rb in cells carrying the R579X mutant.

## Results

### Aminoglycoside G418 induces readthrough of nonsense mutant *RB1*

**Fig 1:**
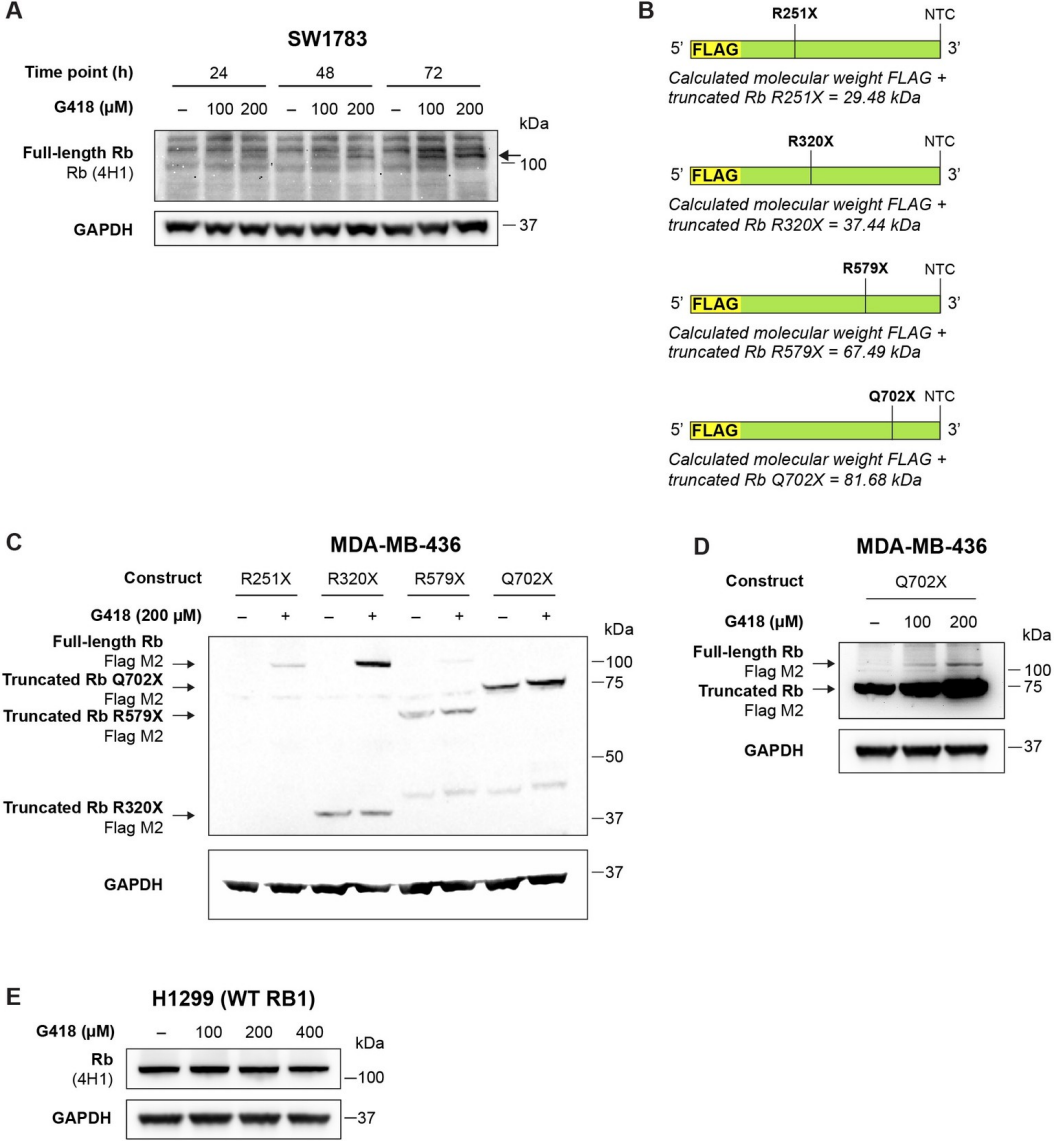
G418 induces readthrough of R251X, R320X, R579X and Q702X nonsense mutant *RB1*. **A,** Western blot analysis of SW1783 cells upon treatment with G418 for 24, 48 or 72 h. The band corresponding to full-length Rb is marked with an arrow. **B,** Plasmid constructs containing an N-terminal FLAG tag followed by the complete *RB1* sequence carrying the R251X (UGA), R320X (UGA), R579X (UGA) or Q702X (UAA) nonsense mutations. Normal termination codons (NTC) in the *RB1* sequence are also indicated. **C** and **D**, Western blot analysis of MDA-MB-436 cells transiently transfected with FLAG-R251X, FLAG-R320X, FLAG-R579X or FLAG-Q702X nonsense mutant *RB1* as indicated and treated with G418 for 72h. **E**, Western blot analysis of H1299 cells carrying WT *RB1* treated with G418 for 72 h. Membranes were first blotted with Rb (4H1) or FLAG (M2) antibody washed and then blotted with GAPDH antibody as loading control.

In order to study readthrough induction of nonsense mutant *RB1* by G418, we used human SW1783 astrocytoma cells that carry endogenous R579X nonsense mutant *RB1* with a UGA PTC. Western blotting revealed induction of full-length Rb in SW1783 cells after 48 and 72 h treatment with G418 (Fig 1A). Maximum induction was observed after 72 hours at 200 μM G418. In order to validate the readthrough ability of G418 for this *RB1* nonsense mutant, we transiently transfected the *RB1*-negative breast carcinoma cell line MDA-MB-436 with a construct containing the complete *RB1* R579X nonsense mutant cDNA with an N-terminal FLAG tag (Fig 1B, see R579X). Treatment with G418 for 72 h resulted in a weak but detectable induction of full-length Rb in these cells according to Western blotting (Fig 1C, see R579X). In order to test readthrough induction of other common *RB1* nonsense mutations with UGA PTCs, MDA-MB-436 cells were also transiently transfected with *RB1* constructs carrying R251X or R320X *RB1* with an N-terminal FLAG tag and treated with G418. As shown in Fig 1C, both nonsense mutants are susceptible to readthrough induction by 200 μM G418. Since it is known that the UGA PTC is more prone to G418-mediated readthrough induction compared to UAG or UAA [16], we also transfected MDA-MB-436 cells with *RB1* cDNA containing the Q702X nonsense mutation with a UAA PTC (Fig 1B) and treated with G418 for 72 h. As compared to the other three mutants, only minor levels of full-length Rb were observed after 200 μM G418 treatment (Fig 1C). The lower part of the gel shown in panel **C** was cut due to the presence of a band in all lanes that cross-reacted with the FLAG antibody. The uncut blot is shown in S1 Fig. A separate experiment confirmed expression of full-length Rb upon treatment with 200 μM G418 for 72 h (Fig 1D).

It is formally possible that the full-length Rb observed in cells carrying nonsense mutant *RB1* after G418 treatment was due to stabilization of full-length Rb protein generated by low levels of basal readthrough rather than induction of translational readthrough. To address this possibility, we examined the effect of G418 on full-length Rb protein in H1299 cells carrying endogenous wild-type (WT) *RB1*. As shown in Fig 1E, G418 had no stabilizing effect on full-length Rb in H1299 cells at the concentrations tested. This argues that the observed increase in full-length Rb protein in cells with nonsense mutant *RB1* was due to genuine translational readthrough rather than mere Rb protein stabilization.

### Readthrough induction of *RB1* R579X is followed by increased *RB1* mRNA levels

**Fig 2:**
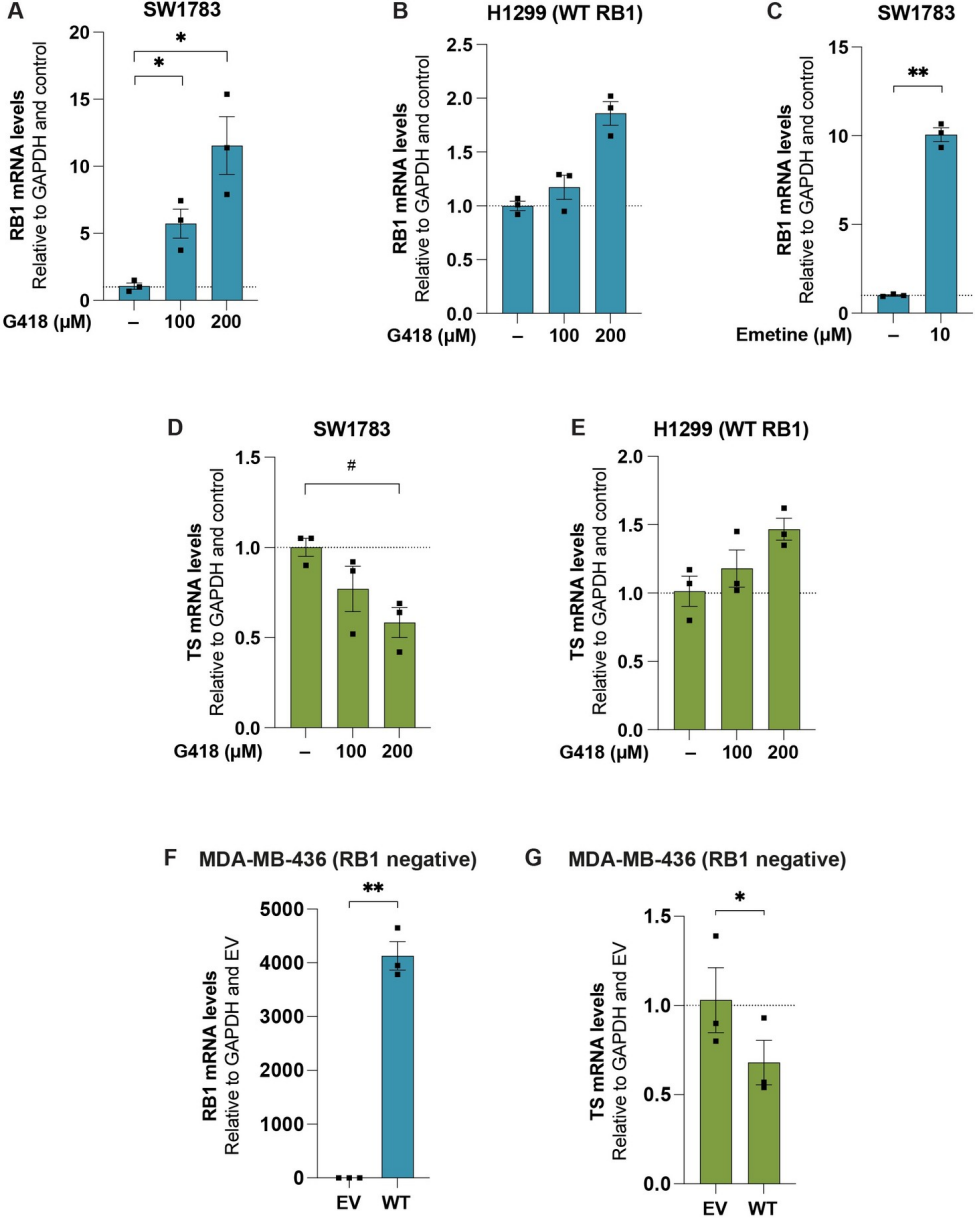
*RB1* expression levels increase and Thymidylate synthase (*TS*) expression levels decrease upon G418 treatment in SW1783 cells. qRT-PCR analysis of *RB1* mRNA in SW1783 **(A)** or H1299 cells **(B)** upon treatment with G418 for 72 h, N = 3. **C**, qRT-PCR analysis of *RB1* mRNA in SW1783 cells upon treatment with Emetine for 6 h, N = 3. DMSO was used as control (-). qRT-PCR analysis of Thymidylate synthase (*TS*) mRNA levels in SW1783 cells **(D)** or H1299 cells **(E)** upon treatment with G418 for 72 h, N = 3. qRT-PCR analysis of *RB1* **(F)** or *TS* **(G)** in MDA-MB-436 upon transfection with empty vector (EV) or WT *RB1* (WT) for 24 h, N = 3. Expression of both genes in each cell line was examined simultaneously with a single GAPDH control; thus, same GAPDH value was used as control for *RB* and *TS*. GAPDH and control samples (-or EV) were used to normalize the gene expression values. Data are represented as mean ± SEM and each dot represents an independent experiment. Statistical analyses were performed comparing each treatment to the control condition (- or EV) and in case of more than two groups, repeated measures one-way ANOVA were used followed by Dunnett’s multiple comparisons test in case of normal data (*p ≤ 0.05) or Friedman test followed by Dunn’s multiple comparisons test (#p ≤ 0.05) was performed in cases where normality could not be proven for these data. In case of two groups comparison, paired t-test was used as data was normal (*p ≤ 0.05, **p ≤ 0.01).

Previous studies have shown that induction of translational readthrough of nonsense mutant *TP53* by G418 increases *TP53* mRNA levels [9, 10]. This has been interpreted as a direct consequence of inhibition of nonsense mediated decay (NMD) which degrades PTC-containing mRNAs. Consistent with these results, we observed not only increased full-length Rb protein levels at increasing concentrations of G418 but also a dose-dependent increase in *RB1* mRNA levels after 72 h treatment in SW1783 cells (Fig 2A). As a control, we performed the same G418 treatment in H1299 cells carrying WT *RB1*. Although *RB1* mRNA levels were modestly induced in these cells at 200 μM G418 (Fig 2B), the increase was only around 1.8 fold, as compared to more than 10-fold increase in the SW1783 cells. To confirm that inhibtion of NMD could indeed lead to elevated *RB1* mRNA levels in SW1783 cells, we treated the cells with the known NMD inhibitor Emetine. We observed a marked increase in *RB1* mRNA upon treatment with Emetine (Fig 2C). Taken together, these results support our conclusion that G418 induces genuine translational readthrough of nonsense mutant *RB1*.

### Thymidylate synthase expression is decreased upon G418 treatment in SW1783 cells

After demonstrating nonsense mutant *RB1* readthrough in SW1783 cells, we aimed to examine if the full-length Rb was active in terms of E2F binding and the consequent effects on E2F target genes at mRNA level in the same cells. We found that the mRNA levels of the E2F target gene thymidylate synthase (*TS*) decreased upon treatment of SW1783 cells with 200 μM G418 (Fig 2D). This is the expected outcome if full-length Rb induced by translational readthrough inhibits E2F transcription activity. Treatment of H1299 WT *RB1* control cells with G418 did not cause any decrease in *TS* mRNA levels, but rather an increase (Fig 2E). Transient transfection of MDA-MB-436 cells with a WT *RB1* construct (Fig 2F) resulted in decreased *TS* mRNA levels, as expected (Fig 2G).

### Combination treatment with G418 and CC-90009 increases readthrough induction of R579X nonsense mutant *RB1*

**Fig 3:**
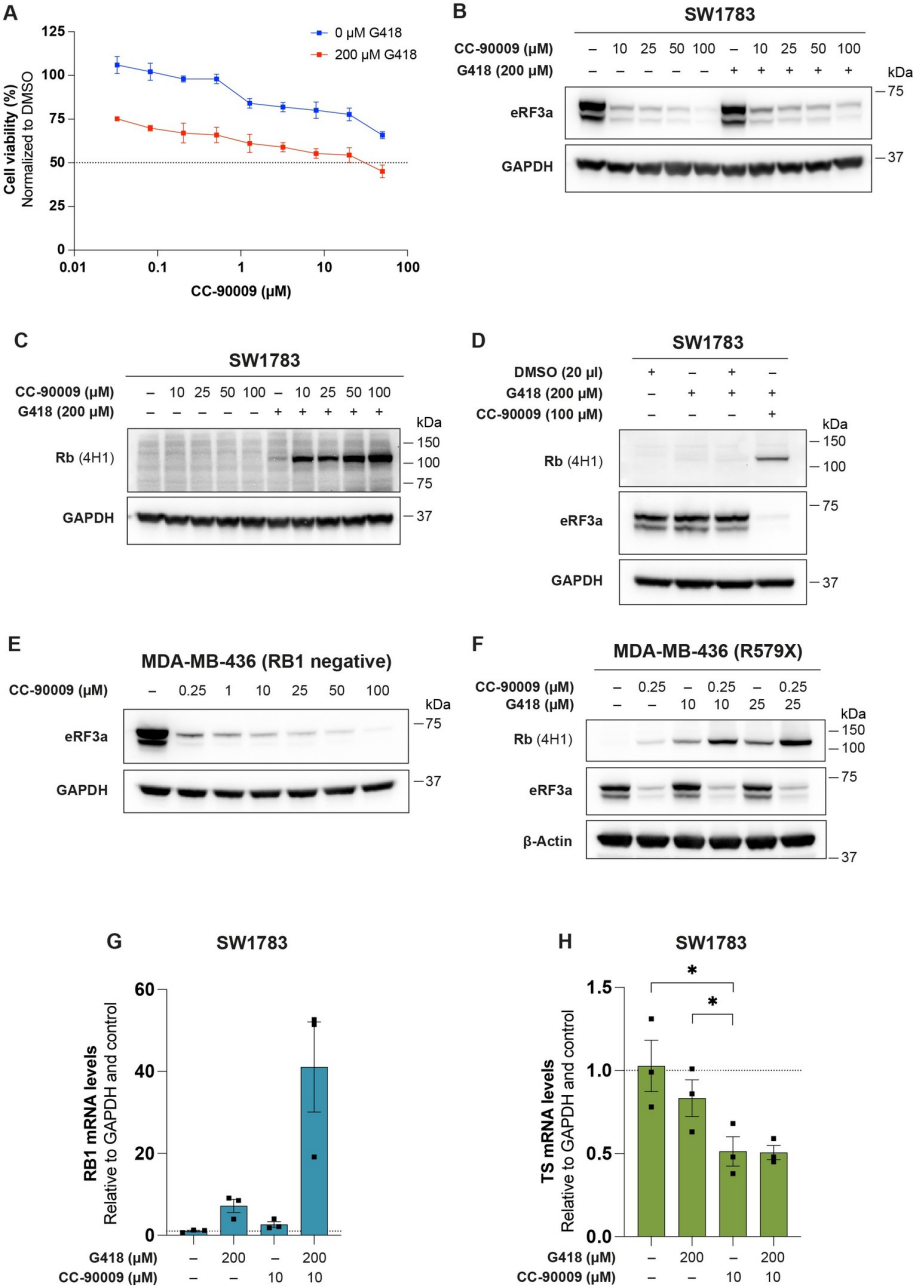
Combination treatment with G418 and CC-90009 potentiates induction of full-length Rb. **A,** Cell viability of SW1783 cells treated with CC-90009 alone (blue line) or combined with G418 (red line) for 72 h as determined by the WST-1 assay, N = 3. Data are represented as mean ± SEM. **B** and **C,** Western blot analysis of eRF3a **(B)** or full-length Rb **(C)** expression in SW1783 cells treated with CC-90009 or G418 alone or in combination for 72 h. Membranes were blotted with Rb (4H1) antibody or eRF3a antibody, washed and then blotted with GAPDH antibody. **D,** Western blot analysis of full-length Rb and eRF3a expression upon treatment with G418 or DMSO alone or G418 combined with DMSO or CC-90009 for 72 h in SW1783 cells. **E,** Western blot analysis of eRF3a expression upon treatment with CC-90009 at different concentrations for 72 h in MDA-MB-436 cells. Membrane was blotted with eRF3a antibody, washed and then blotted with GAPDH antibody. **F,** Western blot analysis of full-length Rb and eRF3a expression upon treatment with CC-90009 or G418 alone or in combination for 72 h in MDA-MB-436 cells transiently transfected with FLAG-tagged R579X mutant *RB1*. **G** and **H**, qRT-PCR analysis of *RB1* **(G)** or *TS* **(H)** mRNA levels in SW1783 cells upon treatment with G418 or CC-90009 alone or in combination for 72 h, N = 3. DMSO was used as control (-). Expression of both genes was examined simultaneously with a single GAPDH control; thus, the same GAPDH value was used as control for *RB* and *TS*. GAPDH and control samples (-) were used to normalize the gene expression values. Data are represented as mean ± SEM and each dot represents an independent experiment. Statistical analyses were performed comparing all conditions to each other using repeated measures one-way ANOVA followed by Tukey’s multiple comparisons test (*p ≤ 0.05). In **D** and **F,** the membranes were blotted with Rb1 (4H1) antibody, washed and blotted with eRF3a antibody, washed and then blotted with GAPDH or β-Actin antibody. In **B, C, D** and **E,** GAPDH was used as loading control. In **F,** β-Actin was used as loading control.

In order to explore possible strategies for potentiating readthrough induction of *RB1* R579X, we performed combination treatment with the known eRF3 degrader CC-90009 [15]. First, cell viability upon single or combination treatment was examined in SW1783 cells using the WST-1 assay. As shown in Fig 3A, treatment with CC-90009 alone had only a modest impact on cell viability and even at 50 μM more than 50% of cells were alive. G418 alone at 200 μM caused a 25% decrease in cell viability, whereas cell viability upon treatment with the combination of 200 μM G418 and 50 μM CC-90009 was around 45%. Fig 3B shows that eRF3a levels were decreased in a dose-dependent manner to a similar extent upon treatment with CC-90009 alone or in combination with G418. As expected, G418 alone did not have any effect on eRF3a levels in SW1783 cells. We then tested the ability of CC-90009 to induce readthrough alone or in combination with G418. Treatment with CC-90009 alone for 72 h did not induce full-length Rb in SW1783 cells at the doses tested, whereas G418 at 200 μM had a weak effect. However, we observed a striking induction of full-length Rb upon combination treatment with 200 μM G418 and increasing concentrations of CC-90009 (Fig 3C).

Since relatively high concentrations of CC-90009 had to be used to demonstrate a decrease in eRF3a in this experiment, the cells were also exposed to higher concentrations of DMSO used as vehicle for CC-90009. To rule out the possibility that the more potent Rb readthrough induction was due to high concentrations of DMSO which might facilitate uptake of G418, a control experiment was performed in which cells were treated with 200 μM G418 with or without 20 μl DMSO, and G418 in combination with CC-90009 as positive control (Fig 3D). DMSO did not further enhance levels of full-length Rb induced by G418 alone, suggesting that the observed increase in full-length Rb was indeed due to the combination of the two compounds and not DMSO *per se*.

In order to further validate these results, combination treatment with G418 and CC-90009 was also performed in MDA-MB-436 cells transiently transfected with R579X nonsense mutant *RB1*. Lower concentrations of both compounds were used as both are more potent in these cells. CC-90009 at 250 nM was sufficient to decrease eRF3a levels (Fig 3E). G418 alone at 10 or 25 μM induced higher levels of full-length Rb than in SW1783 cells (Fig 3F), probably because higher levels of *RB1* mRNA are generated by the strong CMV promoter in the *RB1* cDNA construct. Combination of G418 with CC-90009 increased full-length Rb levels further, although less potently than the same combination treatment in SW1783 cells (Fig 3C and 3F).

Finally, we examined *RB1* and *TS* mRNA levels in SW1783 cells upon combination treatment with G418 and CC-90009. Combination treatment resulted in a marked increase in *RB1* mRNA levels (Fig 3G), consistent with the increase in full-length Rb shown in Fig 3C. However, no further decrease in *TS* mRNA was observed in SW1783 after combination treatment of G418 and CC-90009 as compared to CC-90009 alone (Fig 3H). In H1299 WT *RB1* control cells, *RB1* mRNA levels also increased upon combination treatment as compared to the single treatments (S2A Fig) but not to the same extent as in SW1783 cells. We observed no decrease in *TS* mRNA levels upon combination treatment but rather an increase similar to that observed upon single treatment with G418 (S2B Fig).

## Discussion

The Rb protein encoded by the *RB1* tumor suppressor gene has a key role as regulator of G1 cell cycle progression [1, 2]. Thus, its inactivation by mutation should abrogate normal cell cycle control and allow dysregulated cell growth. *RB1* mutation is a rate-limiting step for development of sporadic and inherited retinoblastoma [17] but also occurs in other types of tumors. Whole genome sequencing demonstrated *RB1* mutations in 14.3% of bladder cancer cases and 8.3% of glioblastoma multiforme cases [18]. *RB1* mutation status has an impact on the treatment response and clinical outcome in various types of cancer [19]. Specifically, lack of functional *RB1*, may lead to increased resistance to hormone therapy, e.g. tamoxifen treatment in breast cancers [20], kinase inhibitor treatment [5] and immunotherapy [21]. Thus, reactivating nonsense mutant *RB1* in such patients could be a promising strategy for improved cancer treatment.

Pharmacological induction of translational readthrough is aimed at restoring expression of full-length proteins from genes that carry nonsense mutations. This approach has been proven to work for several cancer-relevant genes, such as *TP53* [9], *APC* [22] and *PTEN* [12]. However, the clinical use of aminoglycosides is limited as they cause nephrotoxicity [14] and ototoxicity [13]. Nonetheless, the aminoglycoside G418 serves as a good positive control compound for readthrough induction in experimental systems. The molecular mechanism of G418-induced readthrough involves binding to the helix 44 of the 18S ribosomal RNA [23]. Other readthrough inducers have been described, such as SRI-41315, which reduces the eRF1 abundance [24], and 5-Fluorouridine, which is incorporated into mRNA [25]. The compound CC-90009 enhances readthrough induced by G418 by decreasing eRF3a (GSPT1) levels via proteasomal degradation [15]. CC-90009 is a next-generation cereblon E3 ligase modulating agent selective for eRF3a and capable of inducing apoptosis in AML (acute myeloid leukemia) cells [26]. It is currently being tested in clinical trials in patients with relapsed or refractory AML, higher-risk MDS (myelodysplastic syndromes), or AML [26].

Here we show as proof-of-concept that nonsense mutant *RB1* is amenable to readthrough induction by aminoglycoside G418, leading to expression of full-length Rb protein. As observed in the literature, the UGA premature termination codon (PTC) is more prone to readthrough, followed by UAG and UAA [27]. Consistent with this, we detected more efficient readthrough induction of UGA (R251X, R320X and R579X *RB1* mutants) as compared to UAA (Q702X *RB1* mutant).

It has been reported that induction of translational readthrough of an endogenous nonsense mutant gene may result in increased levels of its mRNA, presumably due to decreased nonsense-mediated decay [9, 28]. Indeed, we observed upregulation of *RB1* mRNA levels in SW1783 cells upon treatment with G418, supporting the idea that the induction of full-length Rb is a result of translational readthrough. We also observed a decrease in Thymidylate synthase expression upon G418 treatment in SW1783 cells. This could be related to Rb restoration and inhibition of E2F-mediated transcription, but further studies are needed to confirm this. Moreover, the impact of full-length Rb protein on G1 to S cell cycle transition and cell proliferation should be examined further in appropriate cellular *in vitro* and mouse *in vivo* models to determine whether induction of full-length Rb by translational readthrough can affect the growth of tumor cells that carry nonsense mutated *RB1* in the context of other genetic alterations.

Due to the toxicity associated with G418, strategies to decrease its dose in a therapeutic setting are needed. One attractive possibility is combination treatment with other compounds. We have previously shown that combination of aminoglycosides with p53-Mdm2 inhibitors results in a synergistic increase in full-length p53 protein upon readthrough induction in R213X nonsense mutant *TP53* cells [10]. A more recent study examined the use of CC-90009 and another previously reported cereblon E3 ligase modulator named CC-885 for nonsense suppression therapy [15]. Increased readthrough induction of nonsense mutant *TP53*, among other genes, was observed upon combination treatment with G418 and CC-90009. In agreement with these results, we found that the combination of G418 and CC-90009 led to a striking synergistic increase in the levels of full-length Rb in R579X nonsense mutant *RB1* cells.

In summary, this study shows for the first time that nonsense mutations in the *RB1* tumor suppressor gene are amenable to translational readthrough induction by G418, and reaffirms the promising readthrough effect of combination treatment with G418 and the eRF3 degrader CC-90009. This approach may ultimately open new avenues for the treatment of patients with tumors carrying nonsense mutant *RB1*.

## Materials and methods

### Cells and cell culture

SW1783 astrocytoma cells carrying endogenous R579X nonsense mutant *RB1* (ATCC, USA) were grown in Dulbecco’s Modified Eagle’s Medium low glucose (Sigma-Aldrich/Merck, Germany) supplemented with 10% fetal bovine serum (FBS) (Gibco, USA) when cultured with CO_2_, or in L-15 Medium (Leibovitz) from Sigma-Aldrich/Merck (Germany) supplemented with 10% fetal bovine serum (FBS) (Gibco, USA) when cultured without CO_2_. MDA-MB-436 breast adenocarcinoma cells (a kind gift from Dr. Per Hydbring, Karolinska Institutet, Sweden) and H1299 lung adenocarcinoma cells (ATCC, USA) were grown in RPMI-1640 Medium (Sigma-Aldrich/Merck, Germany) supplemented with 10% FBS (Gibco, USA).

### Drugs and chemicals

Geneticin™ Selective Antibiotic (G418 Sulfate) (50 mg/ml) was supplied as a solution in water (Gibco, USA). CC-90009 (CAS Number: 1860875-51-9) was obtained from Cayman Chemical (USA) and dissolved in Dimethyl sulfoxide (DMSO) from Sigma-Aldrich/Merck (Germany). Emetine (CAS Number: 483-18-1) was obtained from Sigma-Aldrich/Merck (Germany) and dissolved in DMSO.

### Transient transfections

MDA-MB-436 cells were seeded in 6-well plates at 175,000 cells/well in a final volume of 2 ml. Next day, cells were transfected using Lipofectamine 2000 (Invitrogen, USA) according to manufacturer’s recommendations and 0.5 μg of plasmid containing a FLAG tag followed by the full-length *RB1* cDNA carrying the R251X, R320X, R579X or Q702X mutation or WT *RB1* cDNA or empty vector. On the following day, media was changed to remove the excess of plasmid and cells were treated with the indicated compounds for 72 h or harvested directly for analysis. The plasmid construct containing a FLAG-tag followed by the wild-type *RB1* cDNA sequence and the plasmid constructs containing the FLAG-tag followed by the *RB1* sequence carrying R251X or R320X were made by GenScript (USA). Directed mutagenesis was performed on the wild-type *RB1* construct to introduce the R579X (c.1735C to T) and Q702X (c.2104C to T) mutations in the *RB1* gene using the QuikChange II XL Site-Directed Mutagenesis Kit (Agilent, USA) following the manufacturer’s recommendations.

### Western blotting

SW1783 cells were seeded in 6-well plates at 100,000 cells/well, and H1299 at 150,000 cells/well in a final volume of 2 ml. Next day, cells were treated with the indicated compounds for the indicated time points. MDA-MB-436 were seeded at 175,000 cells/well and transfected the following day with the indicated *RB1* cDNA constructs. The next day, media was changed to remove excess of plasmid and cells were treated with the indicated concentrations of G418 and/or CC-90009. Floating and attached cells were harvested using Trypsin-EDTA (Sigma-Aldrich/Merck, Germany) and washed with DPBS (Dulbecco’s Phosphate Buffered Saline) (Sigma-Aldrich/Merck, Germany). Lysis buffer containing 100 mM Tris pH 7.4, 150 mM NaCl and 1% NP40 was used to lyse cells with Protease Inhibitor Cocktail (Sigma-Aldrich/Merck, Germany) added prior to use. Protein quantification was performed using DC™ Protein assay (Bio-rad, USA). Absorbance measurements were performed with Varioskan™ LUX multimode microplate reader (Thermo Scientific, USA). Samples were prepared by diluting the protein extracts in NuPAGE™ LDS Sample Buffer and NuPAGE™ Sample Reducing Agent (both from Thermo Fisher Scientific, USA) and boiling them. Protein samples were loaded into NuPAGE™ 10% Bis-Tris gels and run in NuPAGE™ MOPS SDS Running buffer (both from Thermo Fisher Scientific, USA). Proteins were then transferred to Nitrocellulose Membranes (0.45 μm Pore Size) from Thermo Fisher Scientific (USA) using wet transfer. Wet transfer was performed using NuPAGE® Transfer Buffer (Thermo Fisher Scientific, USA) containing 20% methanol added prior to use and run for 90 min at 100 V at 4°C. Membranes were then blocked with 5% milk in PBS or TBS containing 0.1% Tween-20 (PBS-T or TBS-T) for 1 h at room temperature and blotted for 1 h at room temperature or overnight at 4°C with the primary antibodies anti-Rb (4H1) (#9309) from Cell Signaling used at a 1/2,000 dilution, eRF3a (GSPT1; PA5-62621) from Invitrogen (USA) diluted 1/1,000 or with anti-β-Actin (A5441) from Sigma-Aldrich/Merck (Germany) at a 1/1,000 dilution. The HRP-conjugated secondary antibodies anti-rabbit IgG (65–6120) or anti-mouse IgG (61–6520) both from Invitrogen (USA) and diluted 1/5,000 were used to detect primary antibodies. The following HRP-conjugated antibodies were also used: anti-FLAG M2 antibody A8592 (Sigma-Aldrich/Merck, Germany) used at 1/10,000 dilution, anti-GAPDH antibody 0411 (sc-47724) from Santa Cruz (USA) at a 1/20,000 dilution. SuperSignal™ West Femto Maximum Sensitivity Substrate (Thermo Scientific, USA) was used to visualize proteins in an iBright FL1000 Imaging System (Thermo Fisher Scientific, USA).

### Quantitative real-time PCR (qRT-PCR)

SW1783 cells were seeded at 100,000 to 150,000 cells/well and treated with indicated concentrations of G418 and/or CC-90009 or Emetine. H1299 were seeded at 150,000 cells/well with indicated concentrations of G418 and/or CC-90009. MDA-MB-436 were seeded at 175,000 cells/well and transfected with the WT *RB1* cDNA or empty vector construct on the following day. All treatments for qRT-PCR experiments were performed in 6-well plates. After transfection or treatment as indicated, floating and attached cells were harvested using Trypsin-EDTA (Sigma-Aldrich/Merck, Germany) and washed with DPBS (Sigma-Aldrich/Merck, Germany). RNA was extracted using the RNeasy mini kit (Qiagen, Germany) according to the manufacturer’s recommendations and was quantified using a NanoDrop Spectrophotometer (Thermo Scientific, USA). cDNA synthesis from RNA was performed using the SuperScript II Reverse Transcriptase (Thermo Fisher Scientific, USA). Real-time quantitative PCR was performed in the Applied Biosystems 7500 Real-Time PCR System (Applied Biosystems, USA). TaqMan Gene Expression Assays and FastStart Universal Probe Master (Rox) (Roche, Switzerland) were used. TaqMan probes used were RB1 (Hs01078066_m1), TYMS (Hs00426586_m1) and GAPDH (Hs99999905_m1) (Applied Biosystems, USA). Relative gene expression was calculated by the 2–ΔΔCt method using GAPDH as endogenous control.

### WST-1 cell viability assay

SW1783 cells were seeded in 96-well plates at 3,000 cells/well in a final volume of 100 μl. On the following day, cells were treated with G418 or CC-90009 or in combination or treated with DMSO as a negative control for 72 h. At the treatment endpoint, the cell proliferation reagent WST-1 (Roche, Switzerland) was added to all wells according to manufacturer’s protocol. Absorbance at 450 nm was determined using the Varioskan™ LUX multimode microplate reader from Thermo Scientific (USA).

### Data presentation and statistical analysis

GraphPad Prism version 9.5.0 (GraphPad Software, USA) was used to perform statistical analyses and for data presentation. Data are presented as mean ± standard error of the mean (SEM). Shapiro-Wilk test was used to test normality of the data. If only two groups were compared and the data was normal, paired t-test was performed. If more than two groups were compared and the data was normally distributed, repeated measures one-way ANOVA was applied. If comparing all conditions only to the control sample, Dunnett’s multiple comparisons test was applied. If comparing all conditions to each other, Tukey’s multiple comparisons test was applied. If normality of the data could not be proved and more than two groups were compared, Friedman test followed by Dunn’s multiple comparisons test was performed. Adobe Photoshop 2021 (Adobe, USA) was used for image processing only when needed to improve visualization and applied to the whole image.

## Supporting information

S1 Fig Uncut blot of Fig 1C. (TIF)

S2 Fig Control experiment of combination treatment of G418 and CC-90009 in H1299 (WT *RB1*) cells to examine *RB1* and *TS* mRNA levels. qRT-PCR analysis of *RB1* **(A)** or *TS* mRNA levels **(B)** in H1299 cells upon treatment with G418 or CC-90009 or in combination for 72 h, N = 3. DMSO was used as control (-). Expression of both genes was examined simultaneously with a single GAPDH control; thus, same GAPDH value was used as control for *RB* and *TS*. GAPDH and control samples (-) were used to normalize the gene expression values. Data are represented as mean ± SEM and each dot represents an independent experiment. Statistical analyses were performed comparing each treatment to each other using repeated measures one-way ANOVA followed by Tukey’s multiple comparisons test (*p ≤ 0.05) in case of normal data or Friedman test followed by Dunn’s multiple comparisons test (#p ≤ 0.05) in case where normality could not be proven for the data.
(TIFF)
