# Supplementary figures and images for: Pharmacological induction of translational readthrough of nonsense mutations in the retinoblastoma (RB1) gene
Source: PLoS One. 2023 Nov 2;18(11):e0292468. doi: 10.1371/journal.pone.0292468 (PMC10621805; doi:10.1371/journal.pone.0292468)

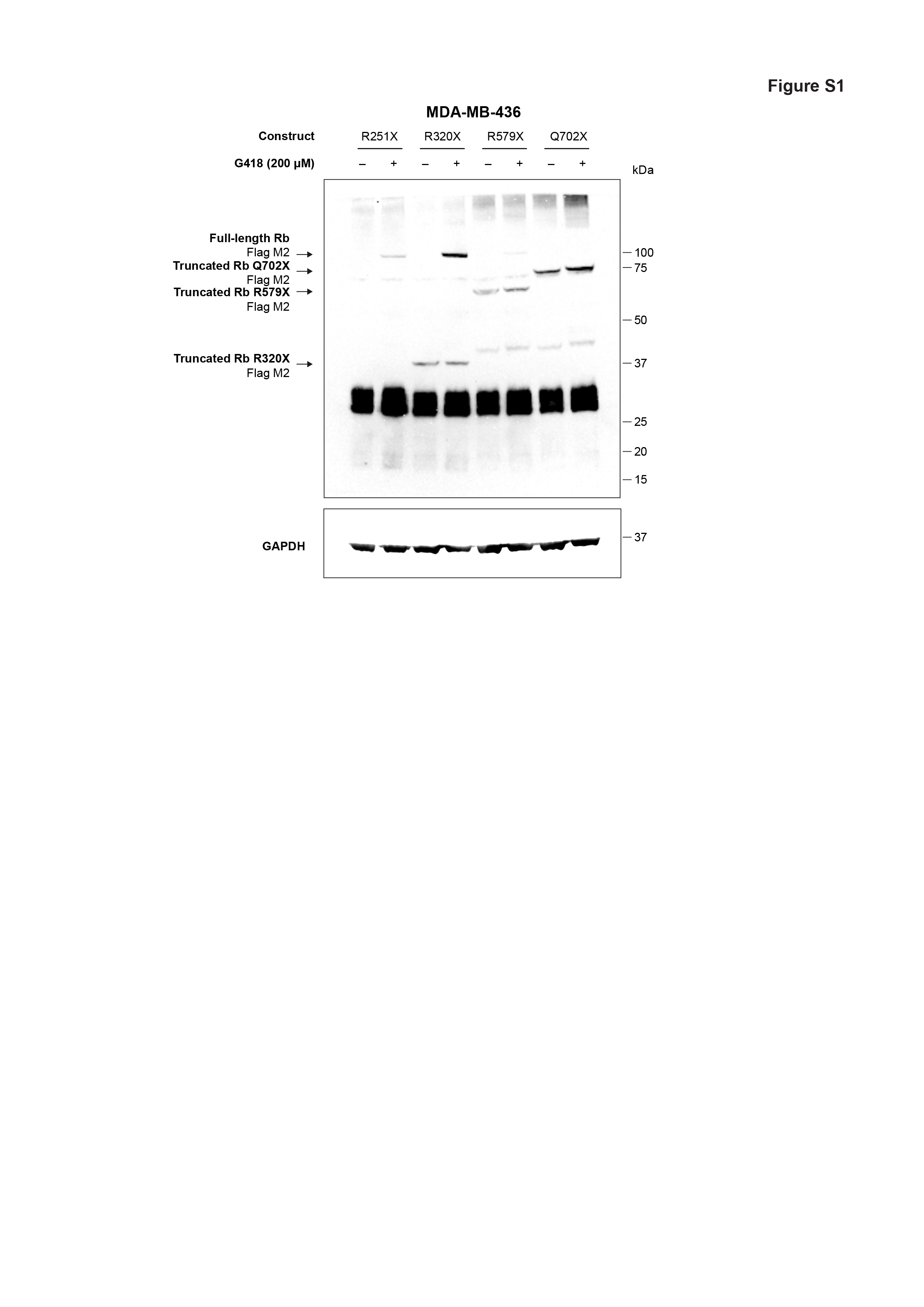

Supplement: S1 Fig — (TIF) [file pone.0292468.s001.tif]

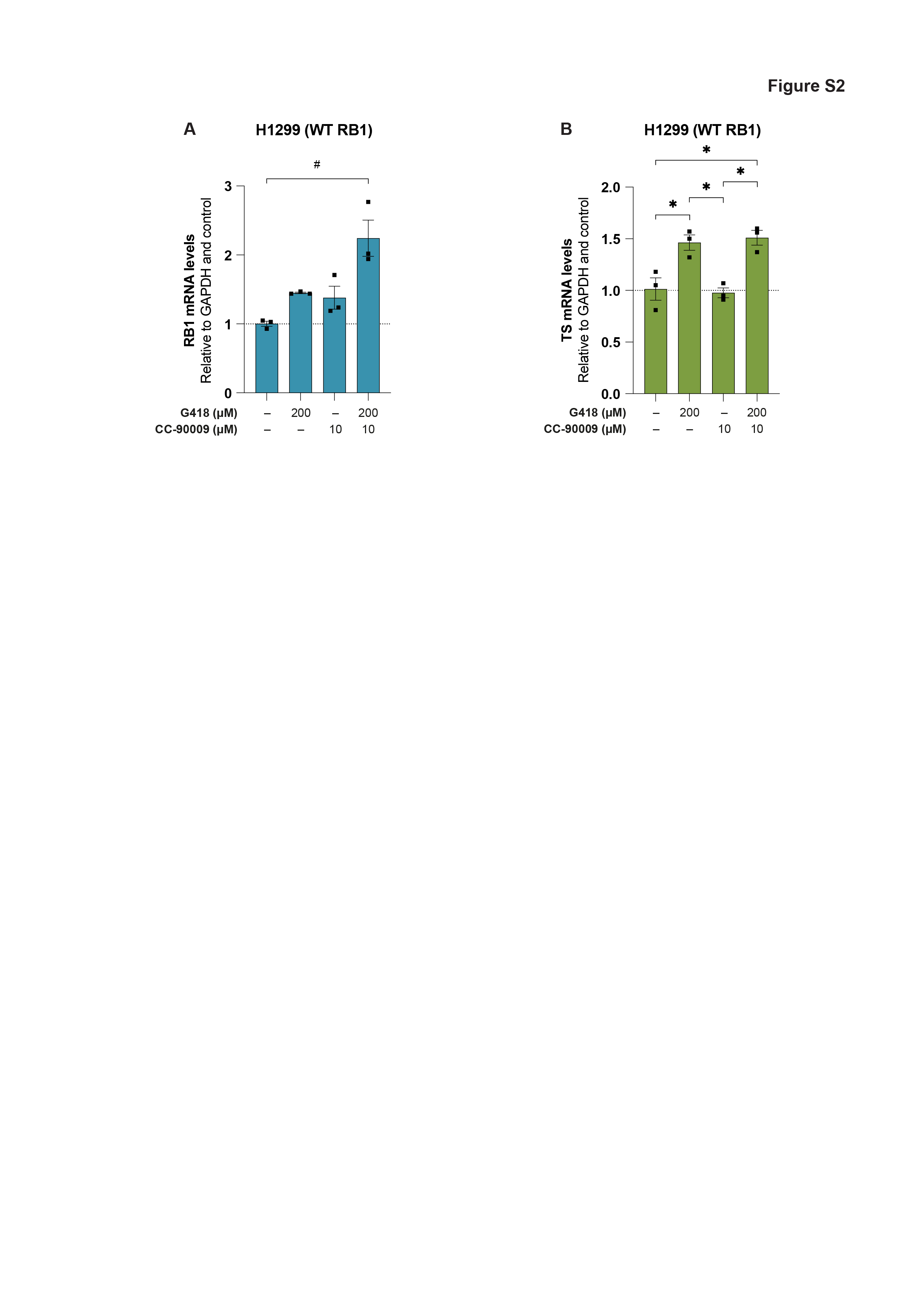

Supplement: S2 Fig — qRT-PCR analysis of RB1 (A) or TS mRNA levels (B) in H1299 cells upon treatment with G418 or CC-90009 or in combination for 72 h, N = 3. DMSO was used as control (-). Expression of both genes was examined simultaneously with a single GAPDH control; thus, same GAPDH value was used as control for RB and TS. GAPDH and control samples (-) were used to normalize the gene expression values. Data are represented as mean ± SEM and each dot represents an independent experiment. Statistical analyses were performed comparing each treatment to each other using repeated measures one-way ANOVA followed by Tukey’s multiple comparisons test (*p ≤ 0.05) in case of normal data or Friedman test followed by Dunn’s multiple comparisons test (#p ≤ 0.05) in case where normality could not be proven for the data. (TIFF) [file pone.0292468.s002.tiff]
